# Tension-Tension Fatigue Behavior of Unidirectional C/Sic Ceramic-Matrix Composite at Room Temperature and 800 °C in Air Atmosphere

**DOI:** 10.3390/ma8063316

**Published:** 2015-06-08

**Authors:** Longbiao Li

**Affiliations:** College of Civil Aviation, Nanjing University of Aeronautics and Astronautics No.29, Yudao St., Nanjing 210016, China; E-Mail: llb451@nuaa.edu.cn; Tel./Fax: +86-25-8489-5963

**Keywords:** ceramic-matrix composites (CMCs), C/SiC, fatigue, S-N curve, hysteresis loops, matrix multicracking, interface debonding

## Abstract

The tension-tension fatigue behavior of unidirectional C/SiC ceramic-matrix composite at room temperature and 800 °C under air has been investigated. The fatigue hysteresis modulus and fatigue hysteresis loss energy corresponding to different number of applied cycles have been analyzed. The fatigue hysteresis loops models for different interface slip cases have been derived based on the fatigue damage mechanism of fiber slipping relative to matrix in the interface debonded region upon unloading and subsequent reloading. The fiber/matrix interface shear stress has been estimated for different numbers of applied cycles. By combining the interface shear stress degradation model and fibers strength degradation model with fibers failure model, the tension-tension fatigue life S-N curves of unidirectional C/SiC composite at room temperature and 800 °C under air have been predicted.

## 1. Introduction

Ceramic materials possess high strength and modulus at elevated temperature. But their use as structural components is severely limited because of their brittleness. The continuous fiber-reinforced ceramic-matrix composites, by incorporating fibers in ceramic matrices, however, not only exploit their attractive high-temperature strength but also reduce the propensity for catastrophic failure. The carbon fiber-reinforced silicon carbide ceramic-matrix composites (C/SiC CMCs) are one of the most promising candidates for many high temperature applications, particularly as aerospace and aircraft thermostructural components [1,2,3].

Compared with ceramic materials and whisker-reinforced CMCs, the fatigue behavior of fiber-reinforced CMCs has many different characteristics, *i.e.*, there exist many matrix microcracks in the fabrication process [4], these microcracks propagated slowly under fatigue loading and matrix failure is not the direct reason for fatigue failure [5]. Ruggles-Wrenn *et al.* [6] investigated the tension-compression fatigue behavior of a SiC/SiC composite at 1200 °C in air. It was found that the tension-compression cycling is much more damaging than tension-tension fatigue, due to more extensive matrix cracking. Under tensile loading, matrix cracks normal to the loading direction; under compression loading, matrix cracks parallel to the loading direction. When matrix cracking and fiber/matrix interface debonding occur, fiber slipping relative to matrix in the interface debonded region leads to interface wear [7,8], *i.e.*, the interface frictional shear stress and fibers strength decrease with the increase of cycle number [9,10,11,12]. Under fatigue loading, fibers fail due to interface wear [13,14]. When the fibers failure probability approaches critical value, the composite would fatigue fracture [15].

In this paper, the tension-tension fatigue behavior of unidirectional C/SiC composite at room temperature and 800 °C under air has been investigated. The fatigue hysteresis modulus and fatigue hysteresis loss energy have been analyzed for different numbers of applied cycles. The fatigue hysteresis loops models corresponding to different interface slipping cases have been derived based on the fatigue damage mechanism of fiber slipping relative to matrix in the interface debonded region upon unloading/reloading. The fatigue hysteresis loss energy for strain energy lost per volume during corresponding cycle is formulated in terms of interface shear stress. By comparing experimental hysteresis loss energy with theoretical computational values, fiber/matrix interface shear stress under fatigue loading has been estimated. The fiber failure probabilities *versus* cycle number curves for different peak stresses are obtained considering interface wear. The tension-tension fatigue life S-N curves have been predicted.

## 2. Material Fabrication and Experimental Procedures

The T-700™ carbon (Toray Institute Inc., Tokyo, Japan) fiber-reinforced silicon carbide matrix composites were provided by Shanghai Institute of Ceramics, People’s Republic of China. The fibers have an average diameter of 7 μm and come on a spool as a tow of 12 K fibers. The unidirectional C/SiC composite was manufactured by hot-pressing method, which offered the ability to fabricate dense composites via a liquid phase sintering method at a low temperature. The volume fraction of fibers was approximately 42%. Low-pressure chemical vapor infiltration was employed to deposit approximately 5–20 layers PyC/SiC with a mean thickness of 0.2 μm in order to enhance the desired non-linear/non-catastrophic tensile behavior. The nano-SiC powder and sintering additives were ball milled for 4 h using SiC balls. After drying, the powders were dispersed in xylene with polycarbonsilane (PCS) to form the slurry. Carbon fiber tows were infiltrated by the slurry and wound to form aligned unidirectional composite sheets. After drying, the sheets were cut to a size of 150 mm × 150 mm and pyrolyzed in argon. Then the sheets were stacked in a graphite die and sintered by hot pressing.

The dog-bone shaped specimens, with dimensions of 120 mm length, 3.2 mm thickness and 4.5 mm width in the gage section, were cut from 150 mm × 150 mm panels by abrasive water jet machining (AWJM) [16,17], in which carborundum abrasive with a size of 150 μm along with a jet pressure of 120 MPa and cutting speed of 100 mm/min. The specimens were further coated with SiC of about 20 μm thick by chemical vapor deposition to prevent oxidation at elevated temperature. These processing steps resulted in a material having bulk density of about 2 g/cm^3^, and an open porosity below 5%.

**Figure 1:**
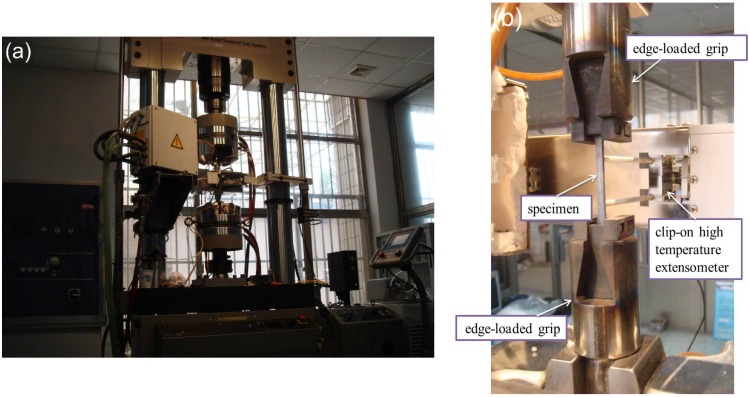
Photograph of fatigue experimental device of unidirectional C/SiC composite (**a**) the MTS Model 809 servohydraulic load-frame; and (**b**) the edge-loaded grips, specimen and clip-on extensometer.

The tension-tension fatigue experiments at room temperature and 800 °C under air were conducted on a MTS Model 809 servo hydraulic load-frame (MTS Systems Corp., Minneapolis, MN, USA) equipped with edge-loaded grips. The fatigue experiments were in a sinusoidal waveform and a loading frequency of 10 Hz to investigate the evolution of stress-strain hysteresis loops and hysteresis loss energy [18,19]. The fatigue load ratio was 0.1, and the fatigue limit was defined to be 1,000,000 cycles. Under fatigue loading, the gage-section strains were measured using a clip-on extensometer (Model No. 634.12F‒24 at room temperature and Model No. 632.53F‒11 at 800 °C, MTS systems Corp.; modified for a 25 mm gage-length), as shown in Figure 1. The tensile fatigue tests were conducted in load control in accordance with the procedure in ASTM standard C 1360 at room and elevated temperatures. The displacement, strain and load were recorded for each cycle. The fatigue hysteresis modulus *E* is calculated by Equation (1).

(1) $$E=\frac{\sigma_{\max}-\sigma_{\min}}{\varepsilon_{\max}-\varepsilon_{\min}}$$

where *σ_max_* and *σ_min_* denote the fatigue peak and valley stresses, respectively; and *ε_max_* and *ε*_min_ denote the fatigue peak and valley strains, respectively.

The direct observations of matrix multicracking at room temperature were made using HiROX optical microscope. The matrix crack densities were determined by counting the number of cracks in a length of about 15 mm. After specimens fatigue failure, the fracture surfaces were observed under optical microscope.

## 3. Experimental Results

### 3.1. Room Temperature

**Figure 2:**
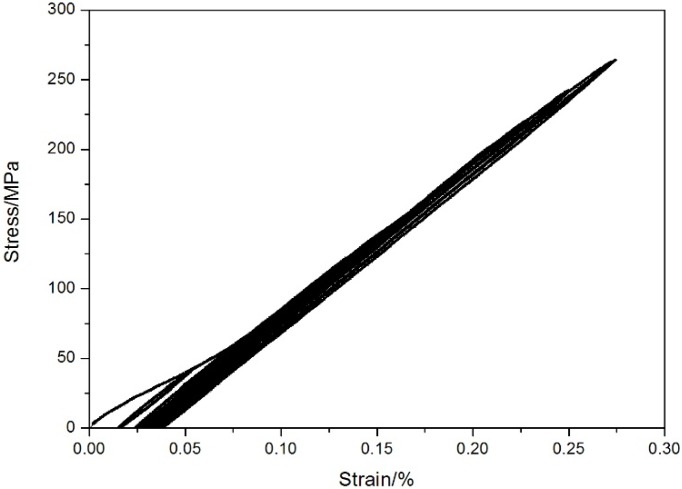
The loading/unloading tensile stress-strain curves of unidirectional C/SiC composite at room temperature.

**Figure 3:**
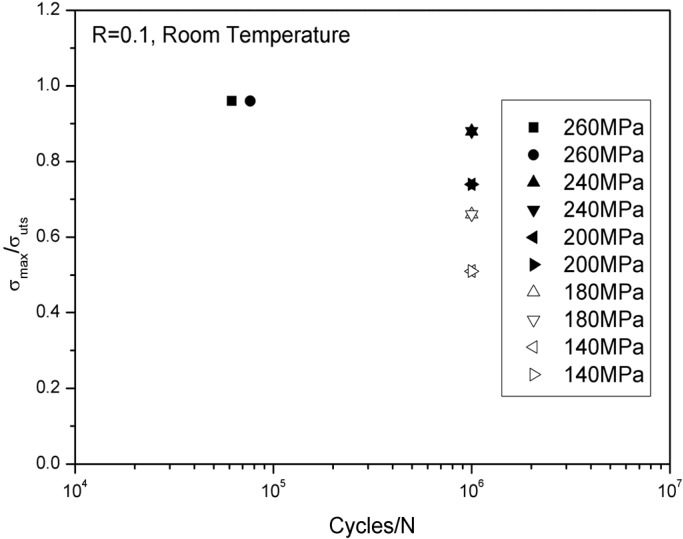
The fatigue life S-N curve of unidirectional C/SiC composite at room temperature.

The monotonic tensile strength is approximately 270 ± 5 MPa at room temperature. The cyclic tensile stress–strain curves are shown in Figure 2. The specimen was loading/unloading/reloading at the peak stresses of 20, 40, 60, 80, 100, 120, 140, 160, 180, 200, 220, 240 and 260 MPa, and failed at the applied stress of 265 MPa, with the failure strain of 0.27%. The tension-tension fatigue peak stresses at room temperature were 260 MPa (96% tensile strength), 240 MPa (88% tensile strength), 200 MPa (74% tensile strength), 180 MPa (66% tensile strength) and 140 MPa (51% tensile strength), respectively. The fatigue life S-N curve at room temperature is illustrated in Figure 3.

**Figure 4:**
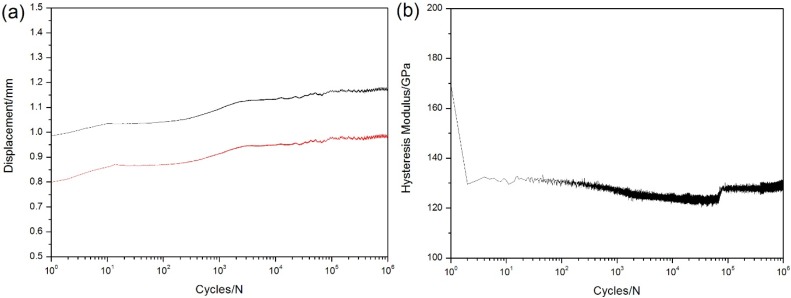
(**a**) The peak and valley displacements *versus* applied cycles; and (**b**) the hysteresis modulus *versus* applied cycles of unidirectional C/SiC composite under *σ*_max_ = 240 MPa at room temperature.

When *σ*_max_ = 240 MPa, the specimen experienced 1,000,000 cycles without fatigue failure. The displacements corresponding to peak and valley stresses increase with the increase of applied cycles, as shown in Figure 4a. Under fatigue loading, the strain and load were recorded for each cycle and the hysteresis modulus *E* is calculated by Equation (1). The hysteresis modulus decreases rapidly at the initial stage of fatigue loading, as shown in Figure 4b, *i.e.*, from 169 to 128 GPa during the first 10 cycles. The occurrence of matrix multicracking and fiber/matrix interface debonding is the main reason for hysteresis modulus degradation during the initial cycles. When matrix cracks approach saturation and interface shear stress degrades to steady-state value, the hysteresis modulus decreases slowly due to fibers failure.

**Figure 5:**
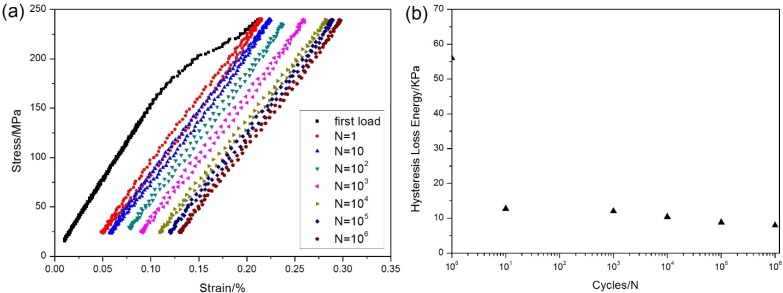
(**a**) The stress-strain hysteresis loops corresponding to different number of applied cycles; and (**b**) the hysteresis loss energy *versus* applied cycles of unidirectional C/SiC composite under *σ*_max_ = 240 MPa at room temperature.

The stress-strain hysteresis loops corresponding to the 1st, 10th, 100th, 1000th, 10,000th, 100,000th and 1,000,000th cycles are illustrated in Figure 5a, and the hysteresis loss energy *versus* applied cycles curve is given in Figure 5b, in which the hysteresis loss energy degrades from 56 KPa during the 1st cycle to 8 KPa during the 1,000,000th cycle due to interface wear.

**Figure 6:**
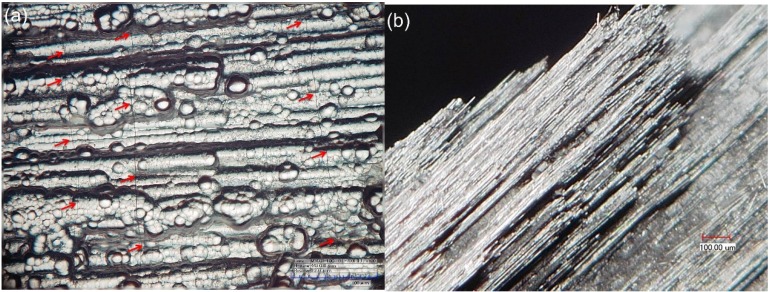
(**a**) The multiple matrix crackings on the side surface; and (**b**) the fiber pullout at the fracture surface of the failure specimen observed under optical microscope.

The specimen without fatigue failure was tensiled to failure at a loading speed of 10 MPa/s. After specimen tensile failure, the side and fracture surface of the failure specimen were observed under optical microscope. There exist multiple matrix cracks on the side surfaces, as shown in Figure 6a. The saturation matrix cracking space was about 108 μm. The fracture surface of the failure specimen is shown in Figure 6b. There exist distributed fiber pullouts. The length of the pullout fiber is much longer than that of tensile failure specimen at room temperature, mainly attributed to low interface shear stress after fatigue loading.

### 3.2. Elevated Temperature

**Figure 7:**
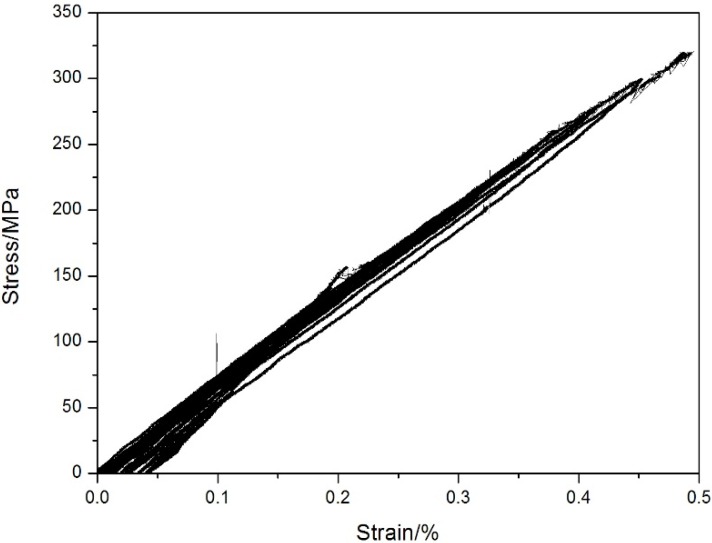
The loading/unloading tensile stress−strain curves of unidirectional C/SiC composite at 800 °C under air.

**Figure 8:**
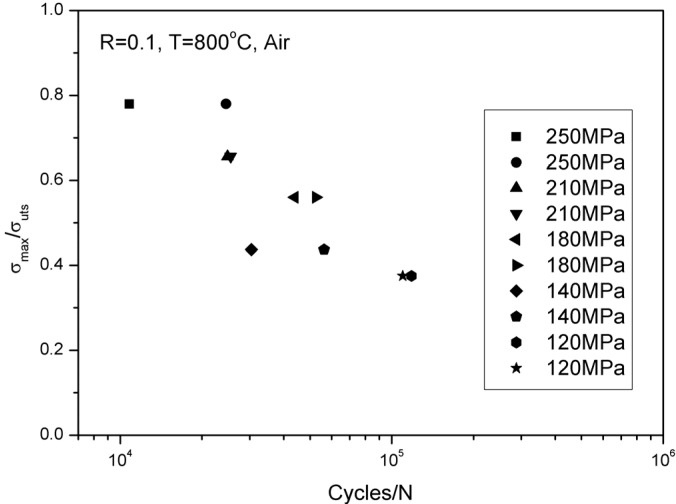
The fatigue life S-N curve of unidirectional C/SiC composite at 800 °C under air.

The tensile strength is approximately 320 ± 3 MPa at 800 °C under air. The cyclic tensile stress-strain curves are shown in Figure 7. The specimen was loading/unloading/reloading at the peak stresses of 20, 40, 60, 80, 100, 120, 140, 160, 180, 200, 220, 240, 260, 280 and 300 MPa, and failed at the applied stress of 320 MPa, with the failure strain of 0.49%. The tension-tension fatigue peak stresses were 250 MPa (78% tensile strength), 210 MPa (65.6% tensile strength), 180 MPa (56% tensile strength), 140 MPa (43.7% tensile strength) and 120 MPa (37.5% tensile strength), respectively. The fatigue life S-N curve is illustrated in Figure 8.

**Figure 9:**
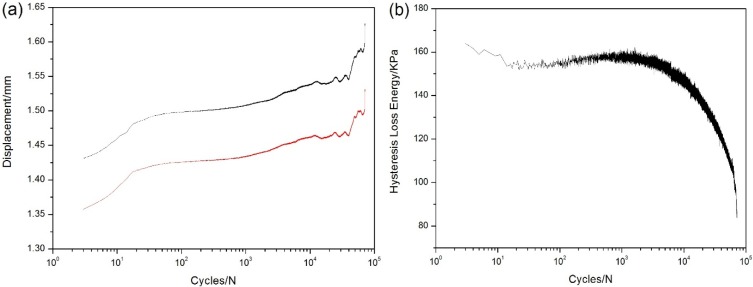
(**a**) The peak and valley displacements *versus* applied cycles; and (**b**) the hysteresis modulus *versus* applied cycles of unidirectional C/SiC composite under *σ*_max_ = 120 MPa at 800 °C in air.

When *σ*_max_ = 120 MPa, the specimen experienced 72215 cycles and fatigue failed. The displacement increases rapidly during the first 10 cycles due to matrix multicracking and interface debonding, then increases slowly due to interface shear stress degradation for interface wear or oxidation, before the specimen failure, the displacement increases rapidly again, mainly attributed to fibers failure, as shown in Figure 9a. The hysteresis modulus *versus* cycle number curve is shown in Figure 9b, in which the curve is divided into three regions, *i.e.*, (1) at the initial stage of fatigue loading, the hysteresis modulus decreases rapidly due to matrix multicracking and interface debonding; (2) when matrix cracks approach saturation, the hysteresis modulus decreases slowly due to interface shear stress degradation; and (3) during the final 2000 cycles, the hysteresis modulus decreases rapidly due to fibers failure.

**Figure 10:**
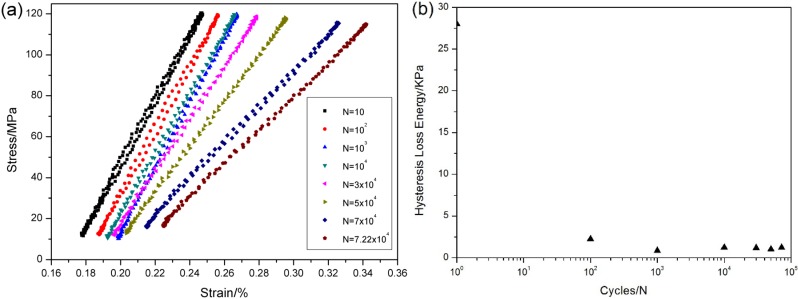
(**a**) The stress-strain hysteresis loops corresponding to different applied cycles; and (**b**) the hysteresis loss energy *versus* applied cycles of unidirectional C/SiC composite under *σ*_max_ = 120MPa at 800 °C in air.

The stress-strain hysteresis loops corresponding to 10th, 100th, 1000th, 10,000th, 50,000th, 70,000th and 72,200th cycles are shown in Figure 10a, and the hysteresis loss energy *versus* applied cycles curve is shown in Figure 10b, in which the hysteresis loss energy degrades from 28 KPa during the 1st cycle to 1.22 KPa during the 72,200th cycle due to degradation of interface shear stress for oxidation.

**Figure 11:**
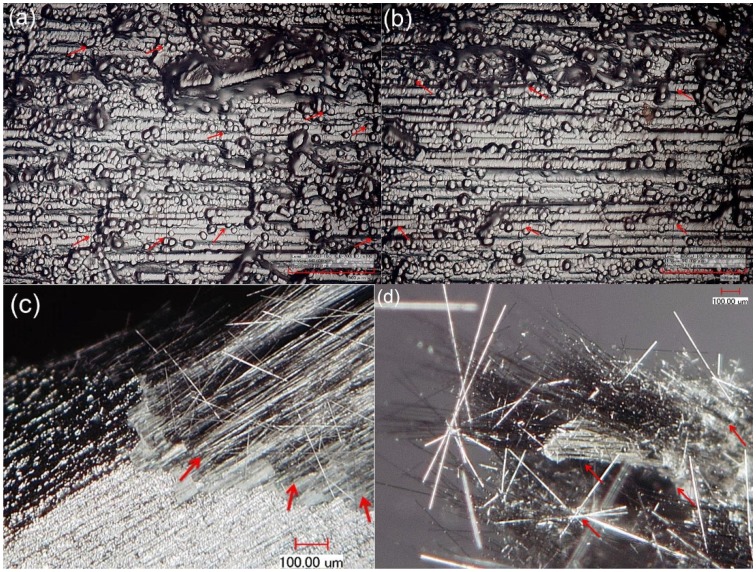
(**a**) The multiple matrix crackings on the front surface; (**b**) the multiple matrix crackings on the side surface; (**c**) the pullout fibers at the fracture surface; and (**d**) the pullout fibers appearing serious oxidation for fatigue failure specimen at 800 °C in air.

After specimen fatigue failure, the front and side surfaces and fracture surface were observed under optical microscope. There exist multiple matrix crackings in front and side surfaces of failure specimens, as shown in Figure 11a,b. The saturation matrix crack spacing is approximately 80 μm. There exist distributed fiber pullouts, and the fiber pullout length is much longer than that of tensile fatigue failure specimen at room temperature, as shown in Figure 11c,d. The surface of pullout fibers exhibits serious oxidation. The interface shear stress degratation due to oxidation of PyC interphase or carbon fibers is the main reason for longer fiber pullout length under fatigue loading at 800 °C in air.

## 4. Fatigue Hysteresis Theories and Life Prediction Model

### 4.1. Fatigue Hysteresis Theories

Under fatigue loading, the hysteresis loops develop due to frictional sliding occurred along any interface-debonded region. Based on the interface slip mechanism, the hysteresis loops can be classified into four different cases, *i.e.*, (1) the interface partially debonds and fiber completely slides relative to matrix; (2) the interface partially debonds and fiber partially slides relative to matrix; (3) the interface completely debonds and fiber partially slides relative to matrix; and (4) the interface completely debonds and fiber completely slides relative to matrix in the interface debonded region.

**Figure 12:**
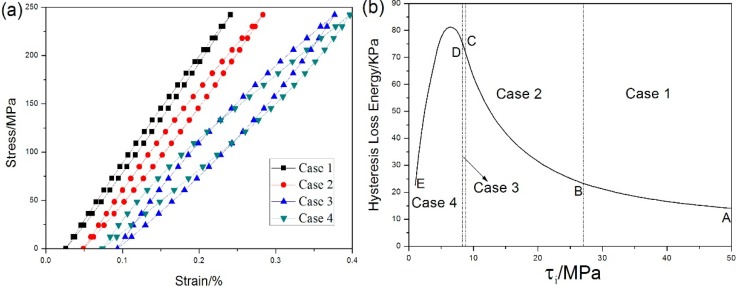

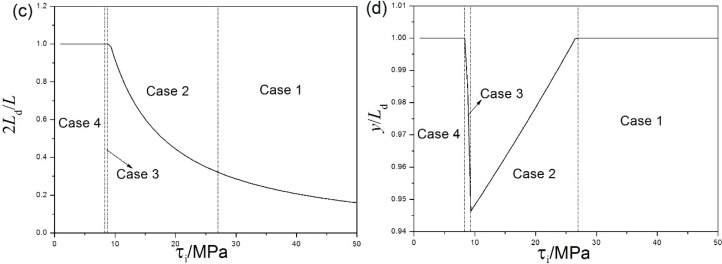
(**a**) The stress-strain hysteresis loops of four interface slip cases; (**b**) the hysteresis loss energy *versus* interface shear stress; (**c**) the interface debonded length 2*L*_d_/*L* *versus* interface shear stress; and (**d**) the interface counter slip length *y*/*L*_d_ *versus* interface shear stress of unidirectional C/SiC composite under *σ*_max_ = 240 MPa.

The shape and area of hysteresis loops as a function of interface shear stress are shown in Figure 12. The stress-strain hysteresis loops corresponding to four different interface slip cases as a function of fiber/matrix interface shear stress under *σ*_max_ = 240MPa are given in Figure 12a, in which the shape and location are different from each other. The hysteresis loss energy as a function of interface shear stress, *i.e.*, τ_i_ = 1–50 MPa, is shown in Figure 12b. When τ_i_ = 27–50 MPa, *i.e.*, the A–B part in Figure 12b, the hysteresis loss energy increases with the decrease of interface shear stress. The hysteresis loops correspond to interface slip Case 1, *i.e.*, the interface partially debonds, *i.e.*, *L*_d_ < *L*/2 in Figure 12c, and fiber completely slides relative to matrix in the interface debonded region, *i.e.*, *y*(*σ*_min_) = *L*_d_ in Figure 12d. When τ_i_ = 8.8–27 MPa, *i.e.*, the B‒C part in Figure 12b, the hysteresis loss energy increases with the decrease of interface shear stress. The hysteresis loops correspond to interface slip Case 2, *i.e.*, the interface partially debonds, *i.e.*, *L*_d_ < *L*/2 in Figure 12c, and fiber partially slides relative to matrix in the interface debonded region, *i.e.*, *y*(*σ*_min_) < *L*_d_ in Figure 12d. When τ_i_ = 8.3–8.8 MPa, *i.e.*, the C‒D part in Figure 12b, the hysteresis loss energy increases with the decrease of interface shear stress. The hysteresis loops correspond to interface slip Case 3, *i.e.*, the interface completely debonds, *i.e.*, *L*_d_ = *L*/2 in Figure 12c, and fiber partially slides relative to matrix in the interface debonded region, *i.e.*, *y*(*σ*_min_) < *L*/2 in Figure 12d. When τ_i_ = 1–8.3 MPa, *i.e.*, the D‒E part in Figure 12b, the hysteresis loss energy increases to the peak value, then decreases with the decrease of interface shear stress. The hysteresis loops correspond to interface slip Case 4, *i.e.*, the interface completely debonds, *i.e.*, *L*_d_ = *L*/2 in Figure 12c, and fiber completely slides relative to matrix in the interface debonded region, *i.e.*, *y*(*σ*_min_) = *L*/2 in Figure 12d. By comparing experimental hysteresis loss energy with theoretical computational value, the interface shear stress can be estimated for different number of applied cycles.

### 4.2. Fatigue Life Prediction Model

Upon first loading to fatigue peak stress, matrix multicracking and interface debonding occur. There exist partial fibers failure in the interface-debonded region and interface-bonded region. Under fatigue loading, fibers fail due to degradation of interface shear stress and fibers strength. When fibers failure probability approaches critical value, the composite would fatigue fail. When fibers begin to break, the loads dropped by broken fibers would be transferred to intact fibers at the cross-section plane. There are two dominant failure criterions presented in the literatures for modeling fibers failure, *i.e.*, the Global Load Sharing criterion (GLS) [15] and Local Load Sharing criterions (LLS) [20]. The two-parameter Weibull model is adopted to describe fibers strength distribution, and the GLS assumption is used to determine the load carried by intact and fracture fibers [15].

(2) $$\frac{\sigma }{V_{\text{f}}}=T\left[1-P\left(T\right)\right]+\langle T_{\text{b}}\rangle P\left(T\right)$$

where <*T*_b_> denotes the load carried by broken fibers; and *P*(*T*) denotes the fibers failure probability.

(3) $$P\left(T\right)=1-\exp\left\{-{\left(\frac{T}{\sigma_{\text{c}}}\right)}^{m_{\text{f}}+1}{\left(\frac{\sigma_{\text{o}}}{\sigma_{\text{o}}\left(N\right)}\right)}^{m_{\text{f}}}\frac{\tau_{\text{i}}}{\tau_{\text{i}}\left(N\right)}\right\}$$

where *m*_f_ denotes the fiber Weibull modulus; and *σ*_c_ denotes the fiber characteristic strength of a length *δ*_c_ of fiber [15].

(4) $$\sigma_{\text{c}}={\left(\frac{l_{\text{o}}\sigma_{\text{o}}^{m_{\text{f}}}\tau_{\text{i}}}{r_{\text{f}}}\right)}^{1/m_{\text{f}}+1},\delta_{\text{c}}={\left(\frac{\sigma_{\text{o}}r_{\text{f}}l_{\text{o}}^{1/m_{\text{f}}}}{\tau_{\text{i}}}\right)}^{m_{\text{f}}/m_{\text{f}}+1}$$

where *l*_o_ is the reference length; *σ_o_* is the fiber reference strength of a length of *l*_o_ of fiber; and *σ*_o_(*N*) is the fiber strength during the Nth cycle. Lee and Stinchcomb [21] found that fibers strength degraded with the increase of applied cycles.

(5) $$\sigma_{\text{o}}\left(N\right)=\sigma_{\text{o}}\left[1-p_{1}{\left(\log N\right)}^{p_{2}}\right]$$

where *p*_1_ and *p*_2_ are empirical parameters. 

Evans *et al.* [13] developed the interface shear stress degradation model.

(6) $$\tau_{\text{i}}\left(N\right)=\tau_{\text{io}}+\left[1-\exp\left(-\omega N^{\lambda }\right)\right]\left(\tau_{\text{imin}}-\tau_{\text{io}}\right)$$

where *τ_i_*(*N*) is the fiber/matrix interface shear stress of Nth cycle; τ_io_ is the initial interface shear stress; τ_imin_ is the steady-state interface shear stress; *N* is the cycle number; and *ω* and *λ* are empirical parameters.

When fibers fracture, fiber stress drops to zero at the break point, and the stress in the fiber builds up through interface shear stress.

(7) $$T_{\text{b}}\left(x\right)=\frac{2\tau_{\text{i}}\left(N\right)}{r_{\text{f}}}x$$

The slip length *l*_f_ required to build the fiber stress up to its previous intact value is given by Equation (8).

(8) $$l_{\text{f}}=\frac{r_{\text{f}}T}{2\tau_{\text{i}}\left(N\right)}$$

The probability distribution *f*(*x*) of the distance *x* of a fiber break from reference matrix crack plane, provided that a break occurs within a distance ±*l*_f_, is [22]

(9) $$\begin{array}{l} f\left(x\right)=\frac{1}{P\left(T\right)l_{\text{f}}}{\left(\frac{T}{\sigma_{\text{c}}}\right)}^{m_{\text{f}}+1}{\left(\frac{\sigma_{\text{o}}}{\sigma_{\text{o}}\left(N\right)}\right)}^{m_{\text{f}}}\frac{\tau_{\text{i}}}{\tau_{\text{i}}\left(N\right)}\\ \times \exp\left\{-\left(\frac{x}{l_{\text{f}}}\right){\left(\frac{T}{\sigma_{\text{c}}}\right)}^{m_{\text{f}}+1}{\left(\frac{\sigma_{\text{o}}}{\sigma_{\text{o}}\left(N\right)}\right)}^{m_{\text{f}}}\frac{\tau_{\text{i}}}{\tau_{\text{i}}\left(N\right)}\right\},x\in \left[0,l_{\text{f}}\right] \end{array}$$

Using Equations (7)–(9), the average stress carried by broken fibers is

(10) $$\begin{array}{l} \langle T_{\text{b}}\rangle =\int_{0}^{l_{\text{f}}}T_{\text{b}}\left(x\right)f\left(x\right)dx\\ =\frac{T}{P\left(T\right)}{\left(\frac{\sigma_{\text{c}}}{T}\right)}^{m_{\text{f}}+1}{\left(\frac{\sigma_{\text{o}}\left(N\right)}{\sigma_{\text{o}}}\right)}^{m_{\text{f}}}\frac{\tau_{\text{i}}\left(N\right)}{\tau_{\text{i}}}\left\{1-\exp\left[-{\left(\frac{T}{\sigma_{\text{c}}}\right)}^{m_{\text{f}}+1}{\left(\frac{\sigma_{\text{o}}}{\sigma_{\text{o}}\left(N\right)}\right)}^{m_{\text{f}}}\frac{\tau_{\text{i}}}{\tau_{\text{i}}\left(N\right)}\right]\right\}\\ -\frac{T}{P\left(T\right)}\exp\left\{-{\left(\frac{T}{\sigma_{\text{c}}}\right)}^{m_{\text{f}}+1}{\left(\frac{\sigma_{\text{o}}}{\sigma_{\text{o}}\left(N\right)}\right)}^{m_{\text{f}}}\frac{\tau_{\text{i}}}{\tau_{\text{i}}\left(N\right)}\right\} \end{array}$$

Substituting Equation (10) into Equation (2), it leads to the form of

(11) $$\frac{\sigma }{V_{\text{f}}}=T{\left(\frac{\sigma_{\text{c}}}{T}\right)}^{m_{\text{f}}+1}{\left(\frac{\sigma_{\text{o}}\left(N\right)}{\sigma_{\text{o}}}\right)}^{m_{\text{f}}}\frac{\tau_{\text{i}}\left(N\right)}{\tau_{\text{i}}}\left\{1-\exp\left[-{\left(\frac{T}{\sigma_{\text{c}}}\right)}^{m_{\text{f}}+1}{\left(\frac{\sigma_{\text{o}}}{\sigma_{\text{o}}\left(N\right)}\right)}^{m_{\text{f}}}\frac{\tau_{\text{i}}}{\tau_{\text{i}}\left(N\right)}\right]\right\}$$

Using Equations (5), (6) and (11), the stress *T* carried by intact fibers at the matrix crack plane can be determined for different peak stresses. Substituting Equations (5) and (6) and the intact fiber stress *T* into Equation (3), the fibers failure probability for different number of applied cycles can be determined. When fibers failure probability approaches the critical value q^*^, the composite would fatigue fail [15].

(12) $$q^{*}=\frac{2}{m_{\text{f}}+2}$$

## 5. Experimental Comparisons

### 5.1. Room Temperature

**Figure 13:**
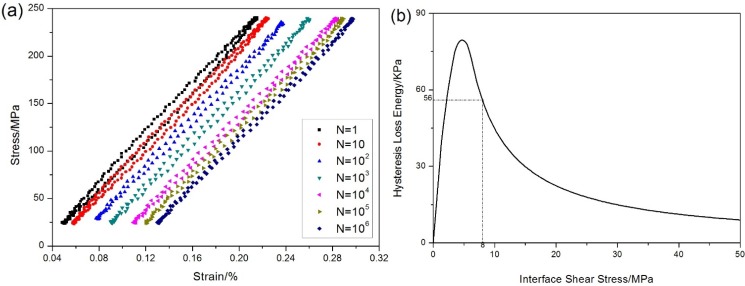
(**a**) The hysteresis loops corresponding to different applied cycles; and (**b**) the hysteresis loss energy as a function of interface shear stress of unidirectional C/SiC composite under fatigue loading at room temperature.

When *σ*_max_ = 240 MPa, the stress-strain hysteresis loops corresponding to the 1st, 10th, 100th, 1000th, 10,000th, 100,000th, 1,000,000th cycles are given in Figure 13a. The hysteresis loss energy as a function of interface shear stress is shown in Figure 13b, in which the experimental hysteresis loss energy of the 1st cycle lies in the right side of the curve. The hysteresis loop of the 1st cycle corresponds to interface slip Case 2, *i.e.*, the interface partially debonds and fiber partially slides relative to matrix in the interface-debonded region upon unloading/reloading. With the increase of number of applied cycles, the interface shear stress degrades due to interface wear. When interface completely debonds, the interface shear stress decreases rapidly due to thermal residual radial tensile stress at the fiber/matrix interface. By comparing experimental hysteresis loss energy with theoretical computational value, the interface shear stress corresponding to different number of applied cycles can be estimated. The predicted interface shear stress of the 1st, 10th, 1000th, 10,000th, 100,000th and 1,000,000th cycles is shown in Table 1, in which the interface shear stress degrades from 8 MPa during the 1st cycle to 0.4 MPa during the 10th cycle.

**Figure 14:**
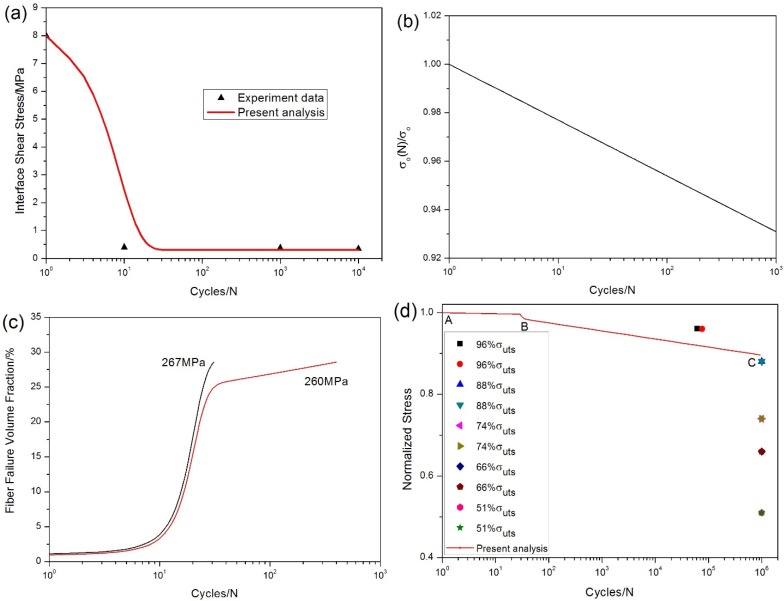
(**a**) the interface shear stress *versus* applied cycles; (**b**) the fibers strength *versus* applied cycles; (**c**) the fibers failure probability *versus* applied cycles; and (**d**) the fatigue life S-N curve of experiment data and present analysis for unidirectional C/SiC composite at room temperature.

The interface shear stress *versus* cycle number has been simulated by Evans-Zok-McMeeking model [13], as shown in Figure 14a, in which the model parameters are given by τ_io_ = 8MPa, τ_imin_ = 0.3MPa, *ω* = 0.04 and *λ* = 1.5. The fibers strength degradation curve predicted by Lee-Stinchcomb model [21] is given in Figure 14b, in which the model parameters are given by *p*_1_ = 0.01 and *p*_2_ = 1.

**Table 1:** The fiber/matrix interface shear stress of unidirectional C/SiC composite corresponding to different number of applied cycles under fatigue loading at room temperature.

Loading cycles	Experimental hysteresis loss energy/KPa	Interface shear stress/MPa
1	56 ± 3	8 ± 0.5
10	12.7 ± 0.5	0.40 ± 0.02
100	12.0 ± 0.5	0.38 ± 0.02
10,000	10.4 ± 0.5	0.35 ± 0.02
100,000	8.8 ± 0.4	0.32 ± 0.02
1,000,000	8.0 ± 0.4	0.30 ± 0.02

The fibers failure probability under *σ*_max_ = 267 and 260 MPa corresponding to different number of applied cycles is illustrated in Figure 14c, in which the fibers failure probability increases with the increase of peak stress, *i.e.*, when *σ*_max_ = 267 MPa, the specimen fatigue failed after 31 cycles; and when *σ*_max_ = 260 MPa, the specimen fatigue failed after 400 cycles. The fibers failure probability *versus* applied cycles curve can be divided into two regions as shown in Figure 14c, *i.e.*, (1) during the first 20 cycles, the fibers failure probability increases rapidly due to degradation of interface shear stress and fibers strength; and (2) when interface shear stress approaches steady-state value, fibers failure is mainly controlled by fibers strength degradation caused by interface wear.

The experimental and predicted fatigue life S-N curves are illustrated in Figure 14d, in which the fatigue limit stress approaches 88% of tensile strength. The predicted fatigue life S-N curve can be divided into two regions as shown in Figure 14d, *i.e.*, (1) the A‒B part is affected by the degradation of interface shear stress and fibers strength; and (2) when interface shear stress approaches steady-state value, the B‒C part is only affected by fibers strength degradation.

### 5.2. Elevated Temperature

**Figure 15:**
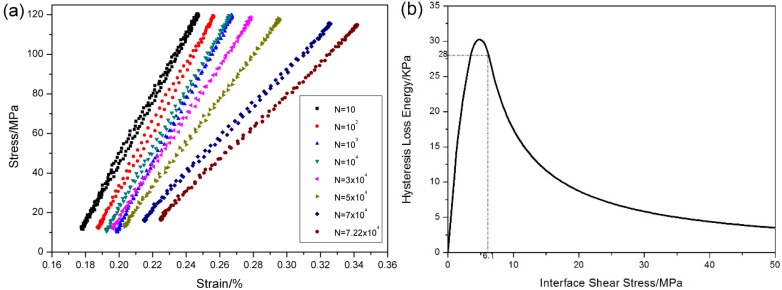
(**a**) The stress-strain hysteresis loops corresponding to different applied cycles; and (**b**) the hysteresis loss energy as a function of interface shear stress of unidirectional C/SiC composite when *σ*_max_ = 120 MPa at 800 °C under air.

When *σ*_max_ = 120 MPa, the stress-strain hysteresis loops corresponding to the 1st, 10th, 100th, 1000th, 10,000th, 30,000th, 50,000th, 70,000th and 72,200th cycles are shown in Figure 15a. The theoretical hysteresis loss energy as a function of interface shear stress curve is given in Figure 15b, in which the experimental hysteresis loss energy of the 1st cycle lies in the right side of the curve. The hysteresis loop of the 1st cycle corresponds to interface slip Case 2, *i.e.*, the interface partially debonds and fiber partially slides relative to matrix in the interface-debonded region upon unloading/reloading. With the increase of applied cycles, the interface shear stress degrades due to oxidation of interphase or carbon fibers. When interface completely debonds, the interface shear stress decreases rapidly due to thermal residual radial tensile stress at the fiber/matrix interface. By comparing experimental hysteresis loss energy with theoretical computational value, the interface shear stress can be estimated for different number of applied cycles. The predicted interface shear stress of the 1st, 100th, 10,000th and 72,200th cycles is illustrated in Table 2, in which the interface shear stress degrades from 6.1 MPa during the 1st cycle to 0.3 MPa during the 100th cycle.

**Table 2:** The interface shear stress of unidirectional C/SiC composite corresponding to different applied cycles when *σ*_max_ = 120 MPa at 800 °C under air.

Loading cycles	Experimental fatigue hysteresis loss energy/KPa	Interface shear stress/MPa
1	28 ± 2	6.1 ± 0.3
100	2.2 ± 0.5	0.30 ± 0.02
10,000	1.4 ± 0.5	0.22 ± 0.02
72,200	1.2 ±0.5	0.20 ± 0.02

**Figure 16:**
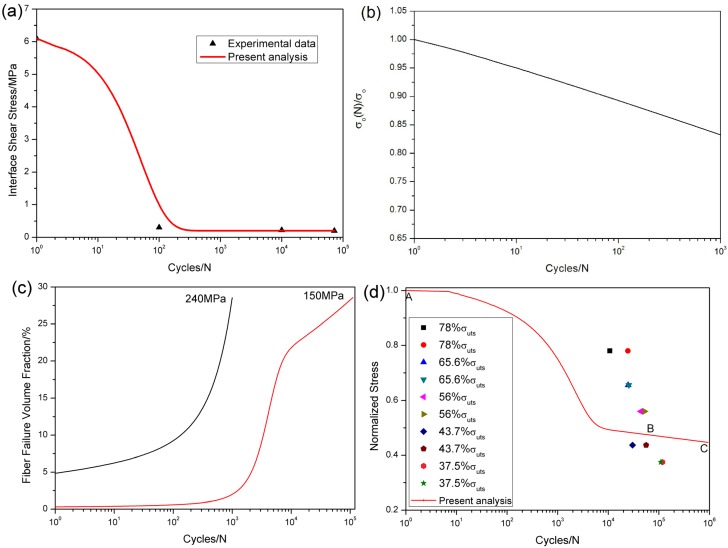
(**a**) the interface shear stress *versus* applied cycles; (**b**) the fibers strength *versus* applied cycles; (**c**) the fibers failure probability *versus* applied cycles; and (**d**) the fatigue life S-N curves of experiment data and present analysis for unidirectional C/SiC composite at 800 °C under air.

The interface shear stress *versus* applied cycles has been simulated by Evans‒Zok‒McMeeking model [13] as shown in Figure 16a, in which the model parameters are given by τ_io_ = 6.1 MPa, τ_imin_ = 0.2 MPa, *ω* = 0.001 and *λ* = 0.8. The fibers strength degradation curve predicted by Lee-Stinchcomb model [21] is shown in Figure 16b, in which the model parameters are given by *p*_1_ = 0.02 and *p*_2_ = 1.

The fibers failure probability corresponding to different number of applied cycles is illustrated in Figure 16c, *i.e.*, when *σ*_max_ = 240 MPa, the specimen fatigue failed after 999 cycles; and when *σ*_max_ = 150 MPa, the specimen fatigue failed after 110,962 cycles. The fibers failure probability *versus* applied cycles curve can be divided into two regions, as shown in Figure 16c, *i.e.*, (1) at the initial stage of fatigue loading, the fibers failure probability increases rapidly due to degradation of interface shear stress and fibers strength; and (2) when interface shear stress approaches steady-state value, fibers failure is mainly controlled by fibers strength degradation by oxidation.

The experimental and predicted fatigue life S-N curves are given in Figure 16d, in which the fatigue life at 800 °C under air is greatly reduced compared with that at room temperature, mainly attributed to oxidation of interphase or carbon fibers. The predicted fatigue life S-N curve can also be divided into two regions, as shown in Figure 16d, *i.e.*, (1) the A‒B part is affected by the degradation of interface shear stress and fibers strength; and (2) the B‒C part is only affected by fibers strength degradation.

## 6. Conclusions

The tension-tension fatigue behavior of unidirectional C/SiC composite at room temperature and 800 °C under air has been investigated.

(1) The hysteresis modulus decreases rapidly at the initial stage of fatigue loading due to matrix multicracking and interface debonding. When matrix cracks approach saturation, the decrease of hysteresis modulus would be mainly attributed to interface wear. However, at 800 °C in air, the hysteresis modulus decreases rapidly compared with that at room temperature, due to oxidation of interphase and carbon fibers.
(2) At room temperature, the hysteresis loss energy degrades from 56 KPa during the 1st cycle to 8 KPa during the 1,000,000th cycle; and the interface shear stress degrades from 8 MPa to 0.4 MPa during the first 10 cycles under *σ*_max_ = 240 MPa; at 800 °C in air, the hysteresis loss energy degrades from 28 KPa during the 1st cycle to 1.22 KPa during the 72,200th cycle; and the interface shear stress degrades from 6.1 MPa to 0.3 MPa during the first 100 cycles under *σ*_max_ = 120 MPa.
(3) The fatigue limit stress at room temperature was approximately 88% of tensile strength. However, there were no apparent fatigue limit stress at 800 °C in air due to oxidation of interphase or carbon fibers. The fatigue failure specimens were observed under optical microscope. The pullout fibers at 800 °C in air exhibit serious oxidation. At elevated temperature, the degradation of fibers strength due to oxidation of carbon fibers leads to the life being greatly reduced compared with that at room temperature.
(4) The tension-tension fatigue life S-N curves at room temperature and 800 °C under air have been predicted. The predicted fatigue life S-N curve can be divided into two regions, *i.e.*, the first part is affected by the degradation of interface shear stress and fibers strength; and the second part is only affected by fibers strength degradation.

## Nomenclature

*σ*_max_: fatigue peak stress;
*σ*_min_: fatigue valley stress;
*L*: matrix crack spacing;
*L*_d_: fiber/matrix interface debonded length;
*y*: unloading interface counter slip length;
*z*: reloading interface new slip length;
τ_i_: fiber/matrix interface shear stress;
*V*_f_: fiber volume fraction;
*T*: load carried by intact fibers;
<*T*_b_>: load carried by broken fibers;
*P*(*T*): fibers failure probability;
*m*_f_: fiber Weibull modulus;
*σ*_c_: fiber characteristic strength;
*r*_f_: fiber radius;
τ_io_: initial fiber/matrix interface shear stress;
τ_imin_: final steady-state fiber/matrix interface shear stress.
